# Home telemonitoring for patients with acute exacerbation of chronic obstructive pulmonary disease: a randomized controlled trial

**DOI:** 10.1186/s12890-016-0321-2

**Published:** 2016-11-22

**Authors:** Andrea Vianello, Massimo Fusello, Lorenzo Gubian, Claudia Rinaldo, Claudio Dario, Alessandra Concas, Claudio Saccavini, Laura Battistella, Giulia Pellizzon, Giuseppe Zanardi, Silvia Mancin

**Affiliations:** 1 Respiratory Pathophysiology Division, University-City Hospital of Padova, Padova, Italy; 2 Local Health Authority of Venezia, Venezia, Italy; 3 Veneto Region Health Information System, Venezia, Italy; 4 Arsenàl.IT, Veneto Research Centre for e-Health Innovation, Treviso, Italy; 5 Division of Pulmunology, City Hospital of Treviso, Treviso, Italy; 6 U.O. Fisiopatologia Respiratoria, Azienda Ospedaliera di Padova, Via Giustiniani, 1, 35128 Padova, Italy

**Keywords:** Telemonitoring, Chronic obstructive pulmonary disease, Health-related quality of life, Hospitalization

## Abstract

**Background:**

Although a number of studies have suggested that the use of Telemonitoring (TM) in patients with Chronic Obstructive Pulmonary Disease (COPD) can be useful and efficacious, its real utility in detecting Acute Exacerbation (AE) signaling the need for prompt treatment is not entirely clear. The current study aimed to investigate the benefits of a TM system in managing AE in advanced-stage COPD patients to improve their Health-Related Quality of Life (HRQL) and to reduce utilization of healthcare services.

**Methods:**

A 12-month Randomised Controlled Trial (RCT) was conducted in the Veneto region (Italy). Adult patients diagnosed with Class III-IV COPD in accordance with the Global Initiative for Chronic Obstructive Lung Disease (GOLD) classification were recruited and provided a TM system to alert the clinical staff via a trained operator whenever variations in respiratory parameters fell beyond the individual’s normal range. The study’s primary endpoint was HRQL, measured by the Italian version of the two Short Form 36-item Health Survey (SF36v2). Its secondary endpoints were: scores on the Hospital Anxiety and Depression Scale (HADS); the number and duration of hospitalizations; the number of readmissions; the number of appointments with a pulmonary specialist; the number of visits to the emergency department; and the number of deaths.

**Results:**

Three hundred thirty-four patients were enrolled and randomized into two groups for a 1 year period. At its conclusion, changes in the SF36 Physical and Mental Component Summary scores did not significantly differ between the TM and control groups [(-2.07 (8.98) vs -1.91 (7.75); *p* = 0.889 and -1.08 (11.30) vs -1.92 (10.92); *p* = 0.5754, respectively]. Variations in HADS were not significantly different between the two groups [0.85 (3.68) vs 0.62 (3.6); *p* = 0.65 and 0.50 (4.3) vs 0.72 (4.5); *p* = 0.71]. The hospitalization rate for AECOPD and/or for any cause was not significantly different in the two groups [IRR = 0.89 (95% CI 0.79–1,04); *p* = 0.16 and IRR = 0.91 (95% CI 0,75 – 1.04); *p* = 0.16, respectively]. The readmission rate for AECOPD and/or any cause was, however, significantly lower in the TM group with respect to the control one [IRR = 0.43 (95% CI 0.19–0.98); *p* = 0.01 and 0.46 (95% CI 0.24–0.89); *p* = 0.01, respectively].

**Conclusion:**

Study results showed that in areas where medical services are well established, TM does not significantly improve HRQL in patients with COPD who develop AE. Although not effective in reducing hospitalizations, TM can nevertheless facilitate continuity of care during hospital-to-home transition by reducing the need for early readmission.

**Trial registration:**

Retrospectively registered on January 2012, ClinicalTrials.gov Identifier: NCT01513980.

## Background

A common, preventable disease characterized by dyspnea, fatigue, and reduced exercise capacity, Chronic Obstructive Pulmonary Disease (COPD) is a leading cause of death and disability in high, middle, and low income countries [1–4]. Despite international standardized guidelines, improved pharmacotherapy, integrated care, and pulmonary rehabilitation, patients with COPD typically worsen over time and experience episodes of acute exacerbation (AE) [1] which are crucially important in patient management in view of their negative effect on Quality of Life (QoL) and prognosis and the fact that they often lead to hospitalization. Exacerbations of COPD are estimated to result in approximately 110,000 deaths and more than 500,000 hospitalizations per year and have been calculated to account for approximately 70% of COPD-related direct medical costs, with over 18 billion spent in direct costs worldwide annually [5–8].

Telemonitoring (TM) (or remote monitoring) involves the use of telecommunication technology to transmit data regarding patients’ vital signs, symptoms, and medications to an operator at a monitoring station who will, in turn, transmit them to a specialist who can recommend treatment variations or new interventions to avoid deterioration in a patient’s medical condition [9]. Although a number of studies have suggested that TM in patients with chronic diseases can be safe, reliable, and cost effective, systematic reviews have reported inconclusive results regarding its impact on the management of COPD patients, and evidence is still insufficient to draw firm conclusions about its clinical and/or cost effectiveness as far as AE is concerned [9–13].

Acute Exacerbations of COPD (AECOPD) is a major public health problem in the geographic area serviced by our hospital, located in the Veneto region (Italy), and led to approximately 6400 hospitalization in 2009 (http://www.ser-veneto.it/). In view of conflicting results on TM utility in COPD patients, the current study set out to clarify the utility of TM in detecting early signs of AE episodes in advanced-stage COPD patients to permit specialists to take timely, appropriate measures to improve patients’ Health-Related Quality of Life (HRQL) and to reduce the utilization of healthcare services. In this trial, TM was integrated into existing clinical services, and monitoring was provided by teams of pulmonary specialists who already knew the patients who were recruited. Both the intervention and control groups had access to the same conventional clinical care: the only difference was that patients in the study group also had TM.

## Methods

A pragmatic, unblinded, parallel-group, two arm, 12-month Randomised Controlled Trial (RCT) was carried out in the Veneto region (Italy); participants were recruited between 1^st^ November 2011 and 31^st^ July 2012. The study was part of the “RENEWING HEALTH” project, a research initiative involving a Consortium of nine European regions. The trial was registered at ClinicalTrials.gov as NCT01513980 (https://clinicaltrials.gov/ct2/show/NCT01513980?term=renewing+health&rank=3) and was approved by the Ethics Committee of all of the participating centres.

All potential participants were provided with written information about the aims and the procedures of the study, and those willing to participate signed informed consent forms.

### Subjects

Adults at the time of discharge from hospital after an AE episode or attending the outpatient Pulmonary Clinics of the City Hospitals of Padova, Treviso, Venice and Verona (Veneto region of Italy) were recruited. Eligibility criteria for the study were: diagnosis of Class III-IV COPD according to the Global Initiative on Obstructive Lung Disease (GOLD) classification [14]; age ≥ 18 years; life expectancy > 12 months according to Multiparametric Prognostic Index (MPI) [15]; and capability of using, alone or assisted, the TM equipment. Exclusion criteria were: concomitant significant lung disease; unwillingness to provide informed consent or to use the TM technology; the negative advice of the general practitioner (GP), and/or other serious social problems, including lack of adequate family support and/or other social support networks.

### Randomisation

Randomisation was performed following standard procedures and checked for incorrect imbalances or meaningful baseline differences in variables using a dedicated algorithm provided by PASS 2008 software that took into account patient’s age and gender [16]. Patients were randomised to the intervention or control groups using a 2:1 allocation; unequal allocation was preferred for ethical reasons as the patients in the TM group was expected to show improvement in HRQL. Each participating center implemented randomization locally using the same methodology.

### Baseline assessment

The baseline characteristics of the TM and control groups are outlined in Tables 1 and 2. The data collected at baseline included the following: demographic, smoking status, level of education, marital and employment status; the modified Medical Research Council (mMRC) dyspnoea score [17]; the COPD Assessment Test (CAT) score [18]; scores on the Hospital Anxiety and Depression Scale (HADS). HADS, which is an extensively validated measure used to assess the severity of symptoms of mood disorders in hospital or primary care patients and in the general population, consisting of two subscales: anxiety and depression [19, 20]. Another measure taken into consideration was the Short Form Health Survey, Italian version 2 (SF36v2) [21]. The SF36 is a generic measure of HRQL commonly used in studies on COPD and consisting of 8 subscales: general health, physical functioning, role function, role emotional, bodily pain, vitality, social functioning, and mental health]. Two summary scores, a physical component summary (PCS) and a mental component one (MCS) can, in addition, be calculated on the basis of the subscale scores [22–25]. The presence of cardiovascular co-morbidity, the patient’s routine medication and long-term use of Oxygen Therapy (LTOT) were also registered. In addition, baseline Forced Expiratory Volume in the first second (FEV_1_), PaO_2_ and PaCO_2_, and Walking Distance (WD), data gathered from a Pulmonary Function Test (PFT), an Arterial Blood Gas (ABG) analysis, and the 6-minute Walking Test (6-min WT), which were all taken at the time the patients were randomised, were registered. The complete battery of testing was carried out at the various centres taking part in the study.

**Table 1:** Socio-demographic data regarding trial participants at study entry

	TM group *n* = 230	Control group *n* = 104	*P*
Age, y [mean (SD)]	75.96 (6.54)	76.48 (6.16)	0.95
Male/Female [No of participants (%)]	164/66	76/28	0.88
BMI, kg/m^2^ [mean (SD)]	26.55 (4.96)	26.24 (4.91)	0.59
Smoking habit (No of participants)			0.55
• Current Smoker	10 (4.35%)	3 (2.88%)	
• Former Smoker	153 (66.52%)	64 (61.54%)	
• Non-Smoker	65 (28.26%)	36 (34.62%)	
• Packs/year [mean (SD)]	42.35 (63.03)	50.54 (90.50)	0.60
Education level [No of participants (%)]			0.89
• No formal schooling/Less than primary school	20 (9.66%)	10 (10.20%)	
• Primary school	109 (52.66%)	53 (54.08%)	
• Secondary school	39 (18.84%)	16 (16.33%)	
• High school	28 (13.53%)	12 (12.24%)	
• College/University	10 (4.83%)	7 (7.14%)	
• Post graduate degree	1 (0.48%)	0 (0.00%)	
Marital status [No of participants (%)]			0.83
• Never married	6 (2.90%)	3 (3.06%)	
• Currently married	139 (67.15%)	68 (69.39%)	
• Separated	1 (0.48%)	1 (1.02%)	
• Divorces	2 (0.97%)	1 (1.02%)	
• Widowed	55 (26.57%)	25 (25.51%)	
• Cohabitating	4 (1.93%)	0 (0.00%)	
Employment status [No of participants (%)]			0.19
• Government employee	6 (2.90%)	4 (4.08%)	
• Non-government employee	10 (4.83%)	7 (7.14%)	
• Self-employed	9 (4.35%)	7 (7.14%)	
• Non-paid	0 (0.00%)	0 (0.00%)	
• Student	0 (0.00%)	0 (0.00%)	
• Homemaker	12 (5.80%)	10 (10.20%)	
• Retired	170 (82.13%)	70 (71.43%)	
• Unemployed	0 (0.00%)	0 (0.00%)	

Data are presented as Numbers or Means (SD)

*TM* telemonitoring

**Table 2:** Clinical and Lung Function Parameters of the trial participants at study entry

	TM group *n* = 230	Control group *n* = 104	*P*
MRC Dyspnea Score [No of participants (%)]			0.64
• Level 1	26 (11.30%)	10 (9.62%)	
• Level 2	60 (26.09%)	32 (30.77%)	
• Level 3	65 (28.26%)	25 (24.04%)	
• Level 4	53 (23.04%)	29 (27.88%)	
• Level 5	26 (11.30%)	8 (7.69%)	
CAT Score [mean (SD)]	15.23 (8.21)	14.00 (6.82)	0.37
HADS Score [mean (SD)]			
• Anxiety	4.68 (3.45)	5.4 (3.35)	0.09
• Depression	5.1 (4.42)	5.48 (4.49)	0.6
SF36v2			
• PCS	38.36 (9.79)	37.33 (9.42)	0.42
• MCS	44.78 (11.29)	44.57 (11.42)	0.88
Cardiovascular comorbidity [No of participants (%)]			0.63
• Hypertension, n	94 (61.04%)	51 (64.56%)	
• Ischemic Heart Disease, n	60 (38.96%)	28 (35.44%)	
Prescribed medication [No of participants (%)]			
• LABA	221 (97.79%)	96 (94.12%)	0.11
• LAMA	197 (87.17%)	88 (86.27%)	1
• Inhaled steroid	192 (83.48%)	80 (76.92%)	0.18
• Systemic steroid	15 (6.52%)	5 (4.81%)	0.36
LTOT [No of participants (%)]	95 (41.30%)	41 (39.42%)	0.76
Pulmonary Function Test [mean (SD)]			
• FEV_1_, L	1.06 (0.59)	1.09 (0.54)	0.99
• FEV_1_, %	41.90 (8.64)	41.87 (8.30)	0.56
Arterial Blood Gas [mean (SD)]			
• PCO_2_, mmHg	44.31 (7.64)	48.85 (9.28)	<0.004
• PO_2_, mmHg	65.65 (9.89)	65.01 (10.98)	0.81
6-min WT, mt [mean (SD)]	263.4 (95.9)	213.1 (109.4)	0.32

Data are presented as Numbers or Means (SD)

*FEV*_*1*_ Forced Expiratory Volume in the first second, *HADS* Hospital Anxiety and Depression Scale, *LABA* Long-Acting β2-Agonist, *LAMA* Long-Acting Muscarinic Antagonist, *LTOT* Long-Term use of Oxygen Therapy, *MCS* mental component summary, *PCS* physical component summary, *TM* telemonitoring, *6-min WT* 6-minute Walking Test

### Trial intervention: telemonitoring

Patients in the intervention group were provided a TM system consisting of a finger pulse-oxymeter (Wrist Clinic ^TM^ – Medical Concierge, Medic 4 all Italia, Italy) and a gateway device for data transmission over a telephone line to a central data management unit located at the Veneto Regional e-Health Centre. It was possible for a patient/proxy to communicate with the trained operators manning the unit from 08:00–18:00 Monday through Friday. At enrollment, the clinical team responsible for that particular patient explained how to use the TM kit and provided self-management education materials. Additional training was provided by the technician who installed the TM kit at the patient’s home.

**Fig. 1:**
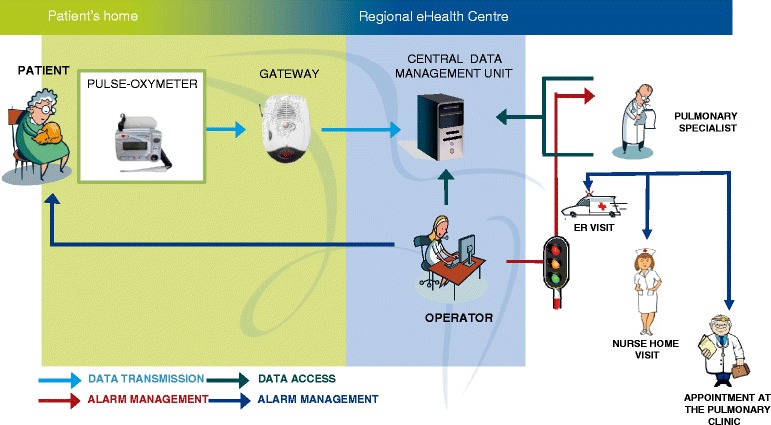
Drawing of how telemonitoring works

Using the TM equipment, patients transmitted their monitored Heart Rate (HR) and Oxygen Saturation (SpO_2_) values to the operator every other day and/or in the event of subjective clinical worsening. A ‘spot check’ or single measurement of SpO_2_ was also performed once a day, usually in the morning. The operators daily (Monday–Friday) reviewed the online data of each patient and if the HR and/or SpO_2_ values that were transmitted were outside of the patient’s “normal” range, they contacted the patient and asked for a second measurement. If the second measurement was also outside of the patient’s normal range, the operator alerted the clinical staff. Values considered out-of-range were customized for every patient depending on his/her individual clinical situation. For patients recruited at discharge from hospital, alarm limits were set on the basis of baseline values obtained from pulmonary function testing carried out before the hospitalization during routine visit with a pulmonary specialist. All patients’ registered data were available to the reference pulmonary specialist on a web-based platform. Once alerted, the specialist him/herself called the patient by telephone to verify if symptoms had stabilized or worsened or if new symptoms had arisen. In the latter event, the patient’s adherence to therapy was checked and, if unsatisfactory, interventions promoting adherence were prescribed. If adherence to treatment proved satisfactory, the diagnosis of AECOPD was confirmed and, the specialist undertook one of the following actions: 1. Modified the patient’s usual medication by telephone. 2. Sent a district nurse (a nurse employed by the National Health Service specialized in making home visits) for a home visit who made a report on the situation. The nurse assessed the subject’s clinical status and adherence to treatment and decided if the patient required an examination by a pulmonary specialist. 3. Set up an office appointment with a pulmonary specialist. 4. Decided that the patient should be taken to the Emergency Department (ED). According to our protocol, the time between when the measurement was outside of the patient’s normal range and the specialist’s recommendation was registered and between when the recommendation was registered and the district nurse or pulmonary specialist actually examined the patient was within 30 min and 48 h, respectively. The specialist made his/her decision on the best course of action depending on the data registered by the TM system, the patients’ symptoms, and his/her clinical judgment. Figure 1 illustrates how the home TM system works.

### Control group

The participants in the two groups received the same clinical care and had access to the same healthcare services. If there were any variations in the clinical status of patients in the control group, he/she directly called/went to see the GP who decided if the patient required an urgent appointment with a pulmonary specialist or a visit to the ED; the former was arranged by the GP. The only difference between the intervention and control groups was that the former also had the TM service. Pharmacologic therapy was provided following international standardized guidelines [14].

### Study end points

The study’s primary endpoint was HRQL, defined as the extent to which the individual’s usual or expected physical, emotional, and social well-being was affected by a medical condition or its treatment [26], which was measured using the Italian version of the SF36v2 [21]. This particular questionnaire was chosen because it covers a broad range of domains of health related issues and seems to capture a subject’s perception of his/her general health; it also acquires a lot of information that is not exclusively related to his/her baseline disease [27].

The study’s secondary end points were:

- Emotional distress, which was measured using the Italian version of the HADS [19];
- the number and duration of hospitalizations due to AECOPD;
- the number and duration of any cause hospitalizations;
- the number of readmissions due to AECOPD. Readmission was defined as a re-hospitalization within 30 days of discharge from hospital due to an AECOPD-related admittance [26].
- the number of any cause readmissions;
- the number of appointments with a pulmonary specialist;
- the number of Emergency Department (ED) visits;
- the number of deaths.

The number and duration of hospitalizations and of readmissions was calculated.

### Data collection

The questionnaires were administered online by a trained operator at baseline and at the end of the 12-month study period. Data on hospital admissions, healthcare service use including consultations with a pulmonary specialist and visits to the ED service, and mortality were extracted from regional records at the end of the trial. The causes of hospital admission were assessed on the basis of the hospital discharge summary. Data were exported to a MySQL database.

### Sample size and statistical analysis

Based on previous studies, we estimated that 196 participants in the intervention group and 99 participants in the control group would be necessary to detect a difference of five points (minimal clinically important difference) in the SF36v2 score between the intervention and control groups at the end of the 1-year study period with 0.5 size effect, 20% drop-out rate, >95% power, using a significance level of 5%. Data analysis was performed on a “per protocol” approach. All the participants who were randomised to the treatment group and began the study were monitored and included in the analysis regardless of their compliance to treatment. Data available concerning deceased patients were included in the analysis. Standard descriptive statistics, including means with standard deviations and/or numbers with associated percentages, were used to analyze the patients’ demographic and clinical characteristics at baseline. Student’s *t*-test or chi-squared test were used to compare continuous or categorical variables, as appropriate. Non-normally distributed variables were compared using the Wilcoxon-Mann-Whitney test. The Shapiro-Wilk test was used to test for data normality. The Central Limit Theorem’s normal approximation was assumed when the SF36 data were assessed since it is a standardized approach used to analyze large patient populations [28]. SF36 scores are expressed as means and standard deviations with a 95% confidence interval (CI) for the difference between two means. Results with regard to hospitalizations, readmissions and visits to the ED are expressed as Incidence Rate per year (IR), Incidence Rate Ratios (IRR) and a 95% CI. IR was calculated by dividing the number of events by the number of days of the study and multiplying the result by 365. Groups were compared using the Poisson test. Deaths are expressed as numbers, percentages, Risk Ratio (RR) and the related 95% CI. Two-tailed *p* values of <0.05 were considered significant for all analyses. All statistical analyses were conducted using the statistical software R version 3.2.2 (Free Software Foundation Inc, Boston, USA).

## Results

### Recruitment

Out of 458 patients originally assessed for eligibility, 334 (73%) were deemed eligible and enrolled in the study and 124 (27%) were excluded; the primary reasons for exclusion were: failure to comply with the eligibility criteria (*n* = 92); decline to participate (*n* = 25); and withdrawal of consent (*n* = 7). The 334 eligible participants were thus randomly assigned to one of two groups: 230 (69%) were assigned to the TM group and 104 (31%) to the control one. Out of the 230 patients allocated to the study group, 19 did not actually participate in the study (and did not receive the TM equipment) for the following reasons: death (*n* = 1), withdrawal of consent (*n* = 9), administrative problems (*n* = 7), moving to a nursing home (*n* = 1), and another reason (*n* = 1). By the end of the one year study period, 30 patients had dropped out of the TM group for the following reasons: death (*n* = 25), withdrawal of consent during the course of the trial (*n* = 3), moving to a nursing home (*n* = 1), and another reason (*n* = 1). At the end of the study, the data of 181 patients who had been randomized to the TM group and actively participated in the study were available for analysis.

**Fig. 2:**
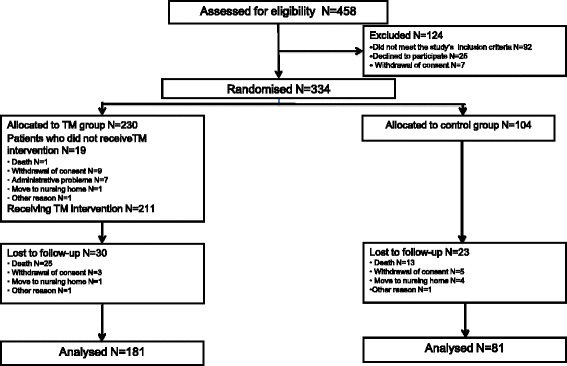
The study’s flow diagram

Out of the 104 patients assigned to the control group, 23 dropped out due to death (*n* = 13), withdrawal of consent during the course of the trial (*n* = 5), moving to a nursing home (*n* = 4), and another reason (*n* = 1). At the end of the study period the data of 81 control patients were analyzed. Figure 2 illustrates the study’s flow diagram.

### Contacts with TM service

There were a total of 2747 TM alerts from the 181 telemonitored patients, meaning an average of 15 contacts per patient over the one year of the trial (approximately one contact every month). No technical problems linked to data transmission between the patients and the e-Health Service were registered.

### Baseline characteristics

The baseline characteristics of the study and control groups are outlined in Tables 1 and 2. There were no differences in the main characteristics of the participants in the two groups, although the controls had slightly worse PaCO_2_ values.

### HRQL

Variations in HRQL are outlined in Table 3. At the end of the study period, changes in the PCS and MCS scores were not significantly different in the two groups [(-2.07 (8.98) vs -1.91 (7.75); *p* = 0.889 and -1.08 (11.30) vs -1.92 (10.92); *p* = 0.5754] and the mean PCS and MCS scores were similar in the two groups (37.11 ± 9.07 vs 36.48 ± 8.64; *p* = 0.59; and 44.56 ± 10.95 vs 43.06 ± 10.95; *p* = 0.31, respectively). There were no significant differences in the scores on the eight subscales of the SF36 between the two groups. The PCS scores at the end of the study were significantly lower with respect to baseline values in both groups (39.18 ± 9.73 vs 37.11 ± 9.07; *p* = 0.002 and 38.39 ± 8.98 vs 36.48 ± 8.64; *p* = 0.02, respectively).

**Table 3:** Participants’ scores on the eight domains and physical and mental component summary scores on the Short Form-36 (S-36) questionnaire at baseline and at the end of the study and differences in the changes between the study and control groups

	TM group	Control group	Difference in the changes in the 2 groups	*P*
Physical function	Baseline: 34.35 (13.34) End of follow-up: 30.92 (12.91) Difference: -3.43 (13.11)	Baseline: 31.88 (12.35) End of follow-up: 29.30 (10.69) Difference: -2.58 (11.02)	−0.85 (-4.16 - 2.44)	0.6105
Role-physical	Baseline: 35.66 (11.79) End of follow-up: 34.58 (11.09) Difference: -1.08 (12.68)	Baseline: 35.36 (12) End of follow-up: 33.16 (9.97) Difference: -2.20 (11.93)	1.12 (-2.16 - 4.42)	0.5006
Bodily pain	Baseline: 50.78 (11.82) End of follow-up: 49.24 (11.75) Difference: -1.54 (13.92)	Baseline: 50.23 (10.8) End of follow-up: 49.04 (12.43) Difference: -1.18 (11.14)	−0.36 (-3.55 - 2.83)	0.8234
General health	Baseline: 37.73 (9.6) End of follow-up: 36.69 (8.34) Difference: -1.04 (9.46)	Baseline: 37.91(8.63) End of follow-up: 36.67 (8.74) Difference: -1.25 (9.19)	0.20 (-2.27 - 2.68)	0.87
Vitality	Baseline: 47.85 (11.04) End of follow-up: 45.67 (9.74) Difference: -2.18 (10.20)	Baseline: 47.44 (9.39) End of follow-up: 43.81 (9.13) Difference: -3.63 (9.27)	1.45 (-1.17 – 4.07)	0.2772
Social functioning	Baseline: 46.09 (11.34) End of follow-up: 44.59 (12.67) Difference: -1.50 (13.74)	Baseline: 45.74 (10.28) End of follow-up: 44.10 (11.28) Difference: -1.64 (12.53)	0.13 (-3.39 – 3.67)	0.9393
Role emotional	Baseline: 35.88 (13.86) End of follow-up: 34.95 (12.36) Difference: -0.94 (14.95)	Baseline: 34.06 (14.08) End of follow-up: 33.67 (13.02) Difference: -0.39 (13.94)	−0.55 (-4.42 – 3.32)	0.7795
Mental health	Baseline: 45.54 (10.77) End of follow-up: 43.80 (10.71) Difference: -1.73 (10.26)	Baseline: 44.87 (11.00) End of follow-up: 41.87 (10.67) Difference: -2.99 (12.85)	1.25 (-1.95 – 4.48)	0.4398
PCS	Baseline: 39.18 (9.73) End of follow-up: 37.11 (9.07) Difference: -2.07 (8.98)	Baseline: 38.39 (8.98) End of follow-up: 36.48 (8.64) Difference: -1.91 (7.75)	−0.16 (-2.44 – -2.12)	0.889
MCS	Baseline: 45.63 (11.22) End of follow-up: 44.56 (10.95) Difference: -1.08 (11.30)	Baseline: 44.98 (10.72) End of follow-up: 43.06 (10.95) Difference: -1.92 (10.92)	−0.84 (-2.11 – 3.80)	0.5754

Data are expressed as Means (SD) and differences (95% CI)

*PCS* physical component summary, *MCS* mental component summary

### Emotional distress

Variations in HADS are outlined in Table 4. At the end of the study period, changes in the “Anxiety” and “Depression” scores were not significantly different in the two groups [0.85 (3.68) vs 0.62 (3.6); *p* = 0.65 and 0.50 (4.3) vs 0.72 (4.5); *p* = 0.71].

**Table 4:** Hospitalisations, appointments with a Pulmonary specialist, visits to the Emergency Department, and deaths during the study period

	TM group	Control group	Difference in the changes in the 2 groups	*P*
HADS Score [mean (SD)]				
• Anxiety	Baseline: 4.68 (3.45) End of f-u: 5.53 (3.47) Difference: 0.85 (3.68)	Baseline: 5.4 (3.35) End of f-u: 6.02 (3.5) Difference: 0.62 (3.6)	0.22 (-0.75 – 1.19)	0.65
• Depression	Baseline: 5.1 (4.42) End of f-u: 5.6 (4.42) Difference: 0.50 (4.3)	Baseline: 5.48 (4.49) End of f-u: 6.2 (4.24) Difference: 0.72 (4.5)	−0.21 (-1.37 -0.94)	0.71
			**IRR**	
Hospitalisations [Incidence Rate per year (95% CI)]				
• Hospitalisations due to AECOPD	0.74 (0.63 – 0.88)	0.84 (0.66 – 1.05)	0.89 (0.79 – 1.04)	0.16
• Hospitalisations for any cause	1.09 (0.95 – 1.25)	1.20 (0.99 – 1.45)	0.91 (0.75 – 1.04)	0.16
• Readmissions due to AECOPD	0.07 (0.04 – 0.11)	0.15 (0.08 – 0.26)	0.43 (0.19 – 0.98)	0.04
• Readmissions for any cause	0.11 (0,07 – 0,16)	0.23 (0.14 – 0.35)	0.46 (0.24- 0.89)	0.01
Duration of hospitalization [bed days; mean(SD)]				
• Hospitalisations due to AECOPD	18.93 (15.33)	23.29 (19.05)	-	0.22
• Hospitalisations for any cause	22.92 (25.11)	25.5 (23.21)	-	0.53
Appointments/Visits [Incidence Rate per year (95% CI)]]				
• Appointments with a Pulmonary specialist	1.41 (1.25 – 1.58)	1.72 (1.46 – 2.01)	0.82 (0.67 - 1)	0.049
• Visits to the Emergency Department	1.29 (1.14 – 1.46)	1.37 (1.14 – 1.63)	0.94 (0.76 – 1.18)	0.58
			RR	
Deaths [No (%)]	23 (11,11)	9 (9,47)	1,17 (0,56 - 2,43)	0.85

Data are expressed as Incidence Rate per year, Means (SD), and Numbers (%)

*f-u* follow-up, *IRR* Incidence Rate Ratio, *RR* Relative Risk

### Hospitalizations, appointments with specialists, and deaths

Data on the utilization of healthcare services are outlined in Table 4. The hospitalization rate per year due to AECOPD and/or to any cause in the TM group was similar to that in the control group [IRR = 0.89 (95% CI 0.79–1.04); *p* = 0.16; and 0.91 (95% CI 0.75 – 1.04); *p* = 0.16, respectively]. The duration of hospitalizations was not significantly different in the two groups. The readmission rate per year due to AECOPD and/or to any cause was significantly lower in the TM than in the control group [IR = 0.07 (95% CI 0.03–0.11) vs 0.15 (95% CI 0.08–0.26); IRR = 0.43 (95% CI 0.19–0.98); *p* = 0.04 and IR = 0.11 (95% CI 0.07–0.16) vs. 0.23 (95% CI 0.14–0.35); IRR = 0.46 (95% CI 0.24–0.89); *p* = 0.01, respectively]. There was a tendency towards fewer specialist examinations at the outpatient pulmonary clinic in the TM with respect to the control group [IR = 1.41 (95% CI 1.25–1.58) vs. 1.72 (95% CI 1.46–2.01); IRR = 0.82 (95% CI 0.67–1); *p* = 0.049]. There was a relatively low mortality rate, and the number of deaths was not significantly different in the two groups [23 (11.11%) vs. 9 (9.47%); RR = 1.17 (95% CI 0.56–2.43); *p* = 0.85)].

## Discussion

The study’s primary finding was that utilization of TM over a one year period had no significant effect on HRQL in the study population. Although TM could theoretically improve HRQL by facilitating early recognition and prompt response to AECOPD, conclusions from previous studies proved controversial. Our results were consistent with those reported by a number of trials suggesting that TM has little effect on HRQL. Nine published studies on home telehealth for COPD evaluated in a systematic review by Polisena et al., in fact, concluded that TM does not improve HRQL when it is compared to usual care [11]. A number of reasons have been advanced to explain the lack of improvement in HRQL and in psychological well-being in TM-monitored patients; these include the burden of self-monitoring, concerns raised by intrusive surveillance, a perceived lack of user-friendliness, and a reduction in the frequency of traditional face-to-face visits [29].

Other studies have, instead, reported that the use of TM can greatly improve HRQL in COPD patients. Bourbeau et al.’s study focusing on a program that included telephone consultations with the treating physician demonstrated a significant improvement in the activity and impact subscales and total scores of St George’s Respiratory Questionnaire (SGRQ) [30]. Likewise, in a RCT conducted by Koff et al. who focused on a TM system similar to ours but including a web-based platform for the transmission of patient’s data and telephone calls by the care coordinator in the event of clinical changes, the TM group showed a significant improvement in the SGRQ score when it was compared with that of the usual care group [31].

Controversial results can at least in part be explained by the considerable heterogeneity in the studies with regard to methodological aspects, such as primary outcome measures, number of participants, disease severity in the study population, and the instruments to measure health status. It is also true that the data obtained in our study based on the summary composite scores of the generic SF36 (physical and mental) are not fully comparable to those utilized by previous trials based on the disease-specific SGRQ. While SGRQ seems to be more sensitive in detecting differences/changes in HRQL linked to the clinical severity of COPD, the SF36 appears to capture additional information that is not exclusively related to the baseline disease, supporting the opinion that the two indexes should be considered complementary [32].

In line with lack of evidence of improved HRQL, TM did not seem to have a positive impact on patients’ emotional distress, in particular on the severity of depression and/or anxiety: indeed, although the aetiology of mood disturbances in COPD patients is multifactoral, HRQL has been considered a stronger determinant with respect to other clinical and/or physiological risk factors [33].

Our healthcare related secondary outcomes did not show that TM had any real effect in preventing AECOPD or other cause-related hospitalisations. A Cochrane systematic review of telehealthcare for COPD including only high-quality evidence from RCTs found, instead, a significant reduction in the number of patients requiring hospital admissions and ED attendances [13]. Most of the programs evaluated had, however, introduced an enhanced clinical service to support the technological arm of the trial that included case management by a nurse as well as other interventions making it difficult to isolate the effect of the TM component. Indeed, our results are consistent with those of a recent RCT by Pinnock et al. who investigated the impact of a TM service that was integrated into existing clinical services for COPD patients. The investigators concluded that it was ineffective in postponing admissions and in improving patients’ QoL and advanced the hypothesis that the positive effect of TM demonstrated in previous studies could have been due to the enhancement of the underpinning clinical service [34].

Some hypotheses explaining why TM did not reduce hospitalization in our study population can be advanced. First, the number of hospital admissions was very low even in the control group [IR: 1,20 (95% CI 0,99–1,45)] and this is not surprising given the health authorities’ efforts to potentiate an integrated care model for chronic COPD patients aiming to provide enhanced long-term home services to reduce need for hospitalization. In this context, there is not much leeway for further reduction in hospitalization as a result of a TM intervention. Second, although variations in HR and SpO_2_ that were used as markers of an unstable clinical condition are regarded as reliable predictors of AECOPD [35], it is reasonable to assume that these physiological parameters cannot always reflect changes in patients’ health status, leading to underestimation and treatment delay of AE episodes. COPD has, in fact, shown high clinical and functional intra-individual variability [36] which may reduce the ability of one-dimensional models to recognize a wide range of events (i.e., worsening dyspnea, reduced number of steps per day, etc) associated to an AECOPD. Third, our trial was not powered for the outcome of hospitalization.

The study produced an interesting result in that it demonstrated that the use of TM was associated with a lower rate of hospital readmission for AECOPD and/or any cause during the first 30 days after hospitalisation [IRR: 0,43 (0,19–0,98); *p* = 0.04 and 0,46 (0,24–0,89); *p* = 0.02, respectively]. In view of the fact that early readmissions are a common occurrence in patients with COPD, which are estimated between 8 and 21.8% of the total number of hospitalizations, and reducing their frequency is considered a high priority of health care organizations [37, 38], study results suggest that TM could be used as an early follow-up care after hospital discharge of COPD patients until the risk of readmission has receded.

A number of limitations pertaining to this study should be mentioned. First, although COPD has been recently regarded as a heterogeneous disease characterized by high phenotype variability [37], phenotypic distinctions in the patients were not considered during the randomization process. In particular, since patients’ history of previous exacerbations was unavailable, we cannot exclude the possibility that the distribution of “frequent exacerbators” was unbalanced between the groups, with a confounding effect on the impact of TM on HRQL. “Frequent exacerbators” have, in fact, been recognized as a distinct clinical subgroup characterized by poorer HRQL as a result of a high exacerbation rate irrespective of the degree of airflow limitation [39]. Second, it was impossible to blind the study population and personnel to treatment allocation, given the intervention’s interactive nature.

## Conclusions

Despite its limitations, the study provides useful information for healthcare professionals who are examining the possibility of utilizing TM to optimize AECOPD management. The study’s most important findings and conclusions can be summarized as follows:

- Adding TM to usual care does not significantly improve patients’ HRQL in a health care context where care standards are high and medical services are well established;
- In this type of situation, TM intervention is not effective in reducing hospitalizations, which is the major factor driving up the healthcare cost of COPD patients;
- TM can nevertheless facilitate continuity of care during the hospital-to-home transition, improving outcomes among patients discharged after an AE episode.

In the light of these findings, we would conclude that use of TM to manage AE should not be generalized across of the entire population of COPD patients and efforts should be made to identify specific subgroups that could most benefit from telehealthcare.

## Abbreviations

6-min WT: 6-minute walking test
ABG: Arterial blood gas
AE: Acute exacerbations
AECOPD: Acute exacerbations of COPD
CAT: COPD assessment test
CI: Confidence interval
COPD: Chronic obstructive pulmonary disease
ED: Emergency Department
FEV_1_: Forced Expiratory Volume in the first second
GOLD: Global initiative for chronic obstructive lung disease
GP: General practitioner
HADS: Hospital anxiety and depression scale
HR: Heart rate
HRQL: Health-related quality of life
IR: Incidence ratio
IRR: Incidence rate ratio
LTOT: Long-term use of oxygen therapy
MCS: Mental component summary
mMRC: modified Medical Research Council
MPI: Multiparametric prognostic index
PaCO_2_: Partial pressure of carbon dioxide in arterial blood
PaO_2_: Partial pressure of oxygen in arterial blood
PCS: Physical component summary
PFT: Pulmonary function test
QoL: Quality of life
RCT: Randomised controlled trial
RR: Risk ratio
SF36v2: Short form health survey version 2
SGRQ: St. George’s Respiratory Questionnaire
SpO_2_: Pulse oximeter oxygen saturation
TM: Telemonitoring
WD: Walking distance
